# VM-UNet++ research on crack image segmentation based on improved VM-UNet

**DOI:** 10.1038/s41598-025-92994-7

**Published:** 2025-03-15

**Authors:** Wenliang Tang, Ziyi Wu, Wei Wang, Youqin Pan, Weihua Gan

**Affiliations:** 1 School of Information and Software Engineering, East China Jiaotong University, Nanchang, 330013 China; 2 School of Transportation and Logistics, East China Jiaotong University, Nanchang, 330013 China

**Keywords:** CNN, Transformer, Mamba, VM-UNet, Crack segmentation, VM-UNet++, Engineering, Mathematics and computing

## Abstract

Cracks are common defects in physical structures, and if not detected and addressed in a timely manner, they can pose a severe threat to the overall safety of the structure. In recent years, with advancements in deep learning, particularly the widespread use of Convolutional Neural Networks (CNNs) and Transformers, significant breakthroughs have been made in the field of crack detection. However, CNNs still face limitations in capturing global information due to their local receptive fields when processing images. On the other hand, while Transformers are powerful in handling long-range dependencies, their high computational cost remains a significant challenge. To effectively address these issues, this paper proposes an innovative modification to the VM-UNet model. This modified model strategically integrates the strengths of the Mamba architecture and UNet to significantly improve the accuracy of crack segmentation. In this study, we optimized the original VM-UNet architecture to better meet the practical needs of crack segmentation tasks. Through comparative experiments on the Crack500 and Ozgenel public datasets, the results clearly demonstrate that the improved VM-UNet achieves significant advancements in segmentation accuracy. Compared to the original VM-UNet and other state-of-the-art models, VM-UNet++ shows a 3% improvement in mDS and a 4.6–6.2% increase in mIoU. These results fully validate the effectiveness of our improvement strategy. Additionally, VM-UNet++ demonstrates lower parameter count and floating-point operations, while maintaining a relatively satisfactory inference speed. These improvements make VM-UNet++ advantageous for practical applications.

## Introduction

Cracks, as one of the common defects on the surface of physical structures, can pose significant safety hazards to the structure if not regularly inspected and repaired, as they may further accumulate and propagate^1^. Currently, there are two main methods for crack detection: one is the traditional manual inspection method^2^, but this method is costly, inefficient, and susceptible to subjective factors, which can lead to missed or incorrect detections; the other is deep learning^3–6^. With the development of deep learning, some researchers have integrated computer vision (CV) tasks into crack detection, achieving efficient and accurate crack detection.

In recent years, the outstanding performance of models based on Convolutional Neural Networks (CNNs)^7^ and Transformer^8^ in visual tasks has not only driven the overall advancement of computer vision technology but also prompted researchers to delve deeper into their exploration in numerous complex visual scenarios, including crack detection.

CNNs have been a significant milestone in the field of computer vision. Early CNNs demonstrated exceptional performance in tasks such as handwritten digit recognition and character classification, establishing their dominance in visual processing. The core advantage of CNNs lies in their unique convolutional kernel design, which captures and integrates key visual information from crack images through local connections and weight sharing. However, due to the inherent locality of CNNs, their ability to capture long-range dependencies is limited, which may result in suboptimal feature extraction and inferior segmentation results.

Transformers were initially developed for natural language processing (NLP) tasks before being introduced to visual tasks. Leveraging their powerful attention mechanism, Transformers have demonstrated unmatched advantages in capturing long-range dependencies in images. As Transformer-based architectures have been widely applied to image processing tasks, their capabilities in vision have been proven, as seen in models like Vision Transformer (ViT)^9^, Swin Transformer^10^, and SegFormer^11^. Although Transformers excel at capturing global context and long-range dependencies, their computational and spatial complexity increases quadratically with the length of the input sequence, which presents an efficiency bottleneck and poses challenges for practical applications. In response, researchers have proposed efficient improvements to Transformer-based models, such as sparse Transformers^12^, linear attention^13^, and FlashAttention^14^. While these models optimize the Transformer architecture by reducing computational and spatial costs without compromising their global perception capabilities, the quadratic complexity issue of Transformers remains unresolved.

The U-Net network^15^ is a classic encoder–decoder architecture. Its U-shaped structure, which combines skip connections between the encoder and decoder and the fusion of features at different levels, enables precise capture of image details and edge information, significantly improving segmentation accuracy and performing excellently in visual tasks. Due to its strong performance in various image segmentation tasks, U-Net has been widely studied and improved. Researchers have proposed several modifications to further enhance its performance in image segmentation tasks, such as U-Net++^16^, which introduces a nested structure with deep supervision mechanisms, ResUNet^17^, which integrates residual learning into its network modules, and Attention U-Net^18^, which strengthens the decoder’s ability to learn features by incorporating an attention gate mechanism.

To leverage the advantages of both Transformer and U-Net architectures, researchers have proposed methods that combine Transformer with U-Net to achieve better performance in image segmentation tasks. For example, models like TransUNet^19^, nnFormer^20^, and Swin-Unet^21^ integrate Transformer modules into the encoder and decoder parts of U-Net to enhance the ability to capture global contextual information. These hybrid models have shown significant advantages in image segmentation tasks, as they combine the global context-awareness of Transformers with the efficient feature fusion mechanism of U-Net. This not only improves segmentation accuracy but also significantly enhances the model’s generalization ability. However, these models also have drawbacks, such as the quadratic computational complexity of Transformers, leading to high computational costs, limited performance improvements on small datasets, and certain missegmentation issues. These shortcomings have prompted researchers to develop new image segmentation architectures that can capture global context information while maintaining linear computational complexity.

Recently, a new model, Mamba^22^, has garnered significant interest among researchers. Mamba is the first foundational model built using a state-space model (SSM). It possesses powerful global modeling capabilities while exhibiting linear computational complexity. This advantage has enabled Mamba to quickly expand across various tasks, such as natural language processing (NLP) and audio modeling. However, due to its design, Mamba is better suited for tasks involving long sequences and autoregressive characteristics, making it less suitable for most visual tasks. In these tasks, Mamba may not fully leverage its advantages, resulting in lower performance compared to traditional CNNs or Transformers. However, with the introduction of Vision Mamba (Vim)^23^ by Zhu et al. and VMamba^24^ by Liu et al., Mamba has successfully been adapted to the computer vision field. Vim and VMamba demonstrate faster processing speeds, as well as lower memory and computational resource requirements, when handling large-scale images and scenarios that demand efficient computation.

Inspired by this, several researchers have introduced Mamba into various image processing tasks for in-depth studies and have effectively deployed it in specific downstream tasks within computer vision (CV). Notably, Mamba has found widespread application in the field of medical image segmentation, with models such as U-Mamba^25^, VM-UNet^26^, Mamba-UNet^27^, and SegMamba^28^. Subsequently, Mamba has also been successfully applied to remote sensing image segmentation, with models like RS3Mamba^29^, CM-UNet^30^, PyramidMamba^31^, and ChangeMamba^32^, demonstrating the powerful capabilities and broad adaptability of Mamba. Recently, Zhao and his team^33^ innovatively applied VM-UNet technology to the fine segmentation of crack images. This attempt marks the first exploration of Mamba’s potential in the field of crack detection, showcasing its significant advantages in this area. The study not only injects new vitality into structural health monitoring but also provides a more efficient and reliable technological solution for infrastructure maintenance and repair.

This study presents innovative improvements and optimizations to the original VM-UNet architecture for the crack segmentation task. A series of innovative designs have been implemented with the aim of enhancing segmentation accuracy. The core feature of this architecture is that, in the encoder stage, we incorporate a dual attention mechanism into the skip connections to enhance the model’s ability to capture and focus on key information, improving its ability to extract and utilize crack features. In the decoder stage, we add a feature fusion module designed to effectively integrate feature information from various stages of the encoder, providing more rich and comprehensive feature inputs to the decoder when reconstructing image details. Through feature fusion, the model can make fuller use of the extracted features, further improving the accuracy of crack segmentation. We expect that our improvements will enhance the performance of VM-UNet in crack segmentation tasks and provide strong support for research and applications in related fields.

The main contributions of this paper are as follows:

1. Improvement of VM-UNet network structure: We have refined the VM-UNet, which was originally designed for crack segmentation tasks, resulting in enhanced effect in this specific task.
2. Introduction of a dual attention mechanism: In the skip connections of VM-UNet, we have introduced a channel and spatial attention mechanism. This dual attention combination enables the model to more accurately focus on key information, improved fracture feature extraction and utilization.
3. Design of a feature fusion module: To further enhance the capabilities of the model, we have designed a feature fusion module that effectively integrates feature information from various stages of the encoder, enriching the feature information available to the decoder. This improves the accuracy and completeness of crack segmentation.
4. The improved VM-UNet performs well in image segmentation tasks, significantly improving the mIoU and mDS metrics. It provides an exploration direction for the further advancement of image segmentation technology.

## Preliminaries

### State space models (SSM)

Gu et al. proposed a new architecture called Mamba, which is based on a selected state space model (SSM). By introducing selective scanning operations and hardware-aware algorithms, it significantly reduces computational complexity. Notably, the Mamba exhibits distinct advantages, characterized by its computational complexity that increases linearly with the length of the input sequence, and its inherent global perception capabilities, which have attracted increasing attention from researchers.

SSM have become an important infrastructure in the field of deep learning, originating from traditional control theory and providing scalability with a linear relationship to the length of data sequences for handling long-term dependencies. Both the structured state space sequence model (S4) and Mamba are based on a continuous time dynamical system that can maps a one-dimensional input function or sequence, denoted as $x\left( t \right) \in R^{L}$, to an output $y\left( t \right) \in R^{L}$ through a series of intermediate hidden states $h\left( t \right) \in R^{L}$. These state space models can be described by the following form of linear ordinary differential equations (ODEs):

1 $$h^{\prime}(t) = Ah(t) + Bx(t)$$

2 $$y(x) = Ch(t) + Dx(t)$$

In state space models, $A \in R^{N \times N}$ is the state matrix, while $B \in R^{N \times 1}$, $C \in R^{N \times 1}$, and $D \in R$ are projection parameters. In order to apply these models to deep learning algorithms, discretization is typically required. Specifically, Δ as a time-scale parameter, it is used to convert the continuous-time parameters A, B into discrete-time parameters $\bar{A}$, $\bar{B}$. The commonly used method for discretization is the zero-order hold (ZOH) rule, which is defined as follows:

3 $$\bar{A} = \exp \left( {\Delta A} \right)$$

4 $$\bar{B} = \left( {\Delta A} \right)^{ - 1} \left( {\exp \left( {\Delta A} \right) - I} \right) \cdot \Delta B$$

After the discretization process, the discrete forms of Eqs. (3) and (4) with step sizes Δ can be reformulated as the following form of recurrent neural network (RNN):

5 $$h^{\prime}_{t} = \bar{A}h_{t - 1} + \bar{B}x_{t}$$

6 $$y_{t} = Ch_{t} + Dx_{t}$$

Furthermore, Eq. (3) can be equivalently transformed into the following form of convolutional neural network (CNN):

7 $$\bar{K} = \left( {C\bar{B},C\bar{A}\bar{B},,C\bar{A}^{L - 1} \bar{B}} \right)$$

8 $$y = x * \bar{K}$$

where $\bar{K} \in R^{L}$ represents a structured convolutional kernel, and L denotes the length of the input sequence x. This convolution method optimizes the calculation process by integrating the output sequence as a whole, which not only accelerates the processing speed but also enhances the model’s adaptability to data of different scales. Moreover, by integrating all elements in the sequence, it enhances the model’s capability to process complex patterns, thereby increasing the overall system’s flexibility and scalability.

## Method

### Overall framework

**Fig. 1:**
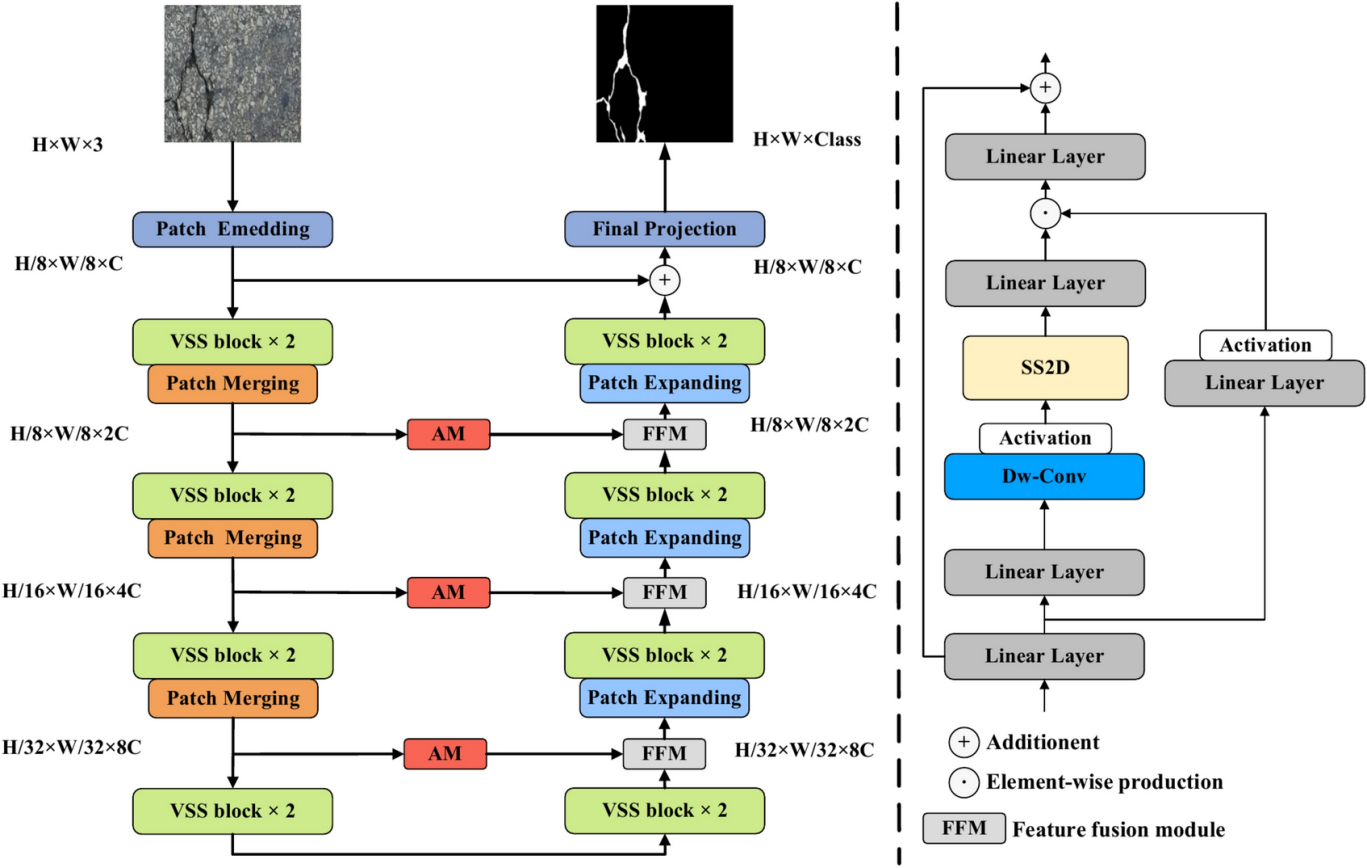
The overall architecture of VM Net++ proposed.

In this section, we introduce the improved VM-UNet, i.e.VM-UNet++. As shown in Fig. 1, in the initial stage, VM-UNet++ employs a patch embedding layer to process the input image data, precisely segmenting the input image into several independent and non-overlapping patches of size 4 × 4, and then transforming the image dimensions to C. The embedded images are then fed into the encoder section, which consists of four core stages for deep feature extraction. In the first three stages, patch merging downsampling modules are adopted to reduce the size of the feature map while increasing its channel count, thus achieving more efficient and precise capture and encoding of image information. Similarly, the decoder section is divided into four stages, applying patch expansion upsampling modules to reduce the number of channels while expanding the feature map size.

The visual state space (VSS) in both the encoder and decoder serves as the core of VM-UNet++, playing a crucial role in feature processing. After layer normalization, the input features are separated into two processing paths. The first path undergoes a linear transformation followed by activation using the Sigmoid linear unit (SiLU)^34^ to enhance feature representation. In the second path, the input features are first transformed by a linear layer, optimized by a depthwise separable convolutional layer, and activated with SiLU. These processed input features then pass through the SS2D module and layer normalization to extract additional features. Finally, the feature outputs from both paths are fused through element-wise operations, processed by a linear layer, and combined with the original residual connection to produce the final output.

In the lateral connections between the encoder and decoder, we innovatively employ channel attention and spatial attention mechanisms. The introduction of these two attention mechanisms enables the model to focus more precisely on key information in the image, thereby improving the accuracy and efficiency of feature extraction. Furthermore, in the decoder section, we enhance the model’s capabilities by adding a feature fusion module. This module effectively fuses feature information from different stages of the encoder, providing a richer source of information for the decoder during image detail reconstruction. This improvement ensures that the model can more comprehensively utilize the extracted features, further enhancing the accuracy and completeness of crack segmentation.

### Attention model

**Fig. 2:**
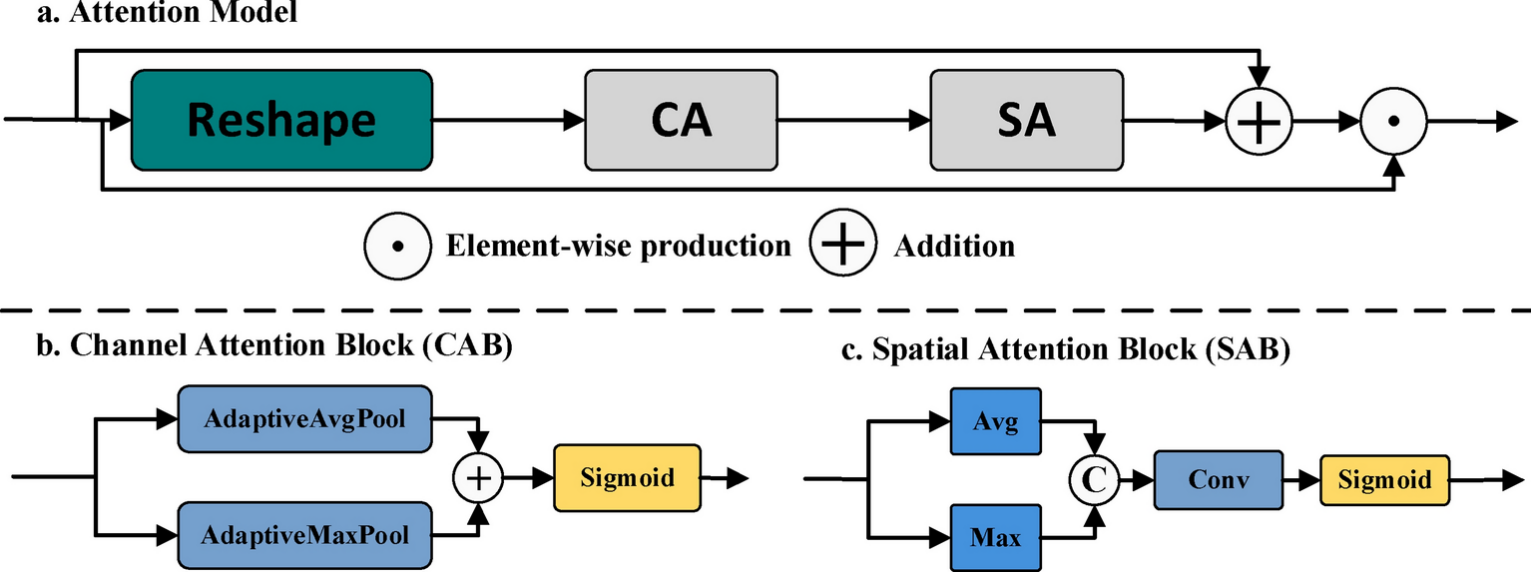
Provides an overview of our attention module. (**a**) Attention module overall network structure, (**b**) Channel attention block, (**c**) Spatial attention block.

We have designed and implemented an efficient attention module to enhance the model’s ability to extract and utilize crack features. As shown in Fig. 2a, the attention module has finely processed and optimized the image features.

First, we use Reshape to adjust the dimensions of the input feature map, ensuring the smoothness and efficiency of data in the subsequent processing steps, laying the foundation for deeper feature extraction. Subsequently, a dual attention mechanism is introduced, consisting of channel attention (CA) and spatial attention (SA). CA emphasizes channels that are more critical for the crack segmentation task by weighting the features of different channels, optimizing feature selection and utilization. SA further enhances the module’s spatial feature extraction capabilities, precisely locating key regions in the image, allowing the model to focus more on the detailed features of cracks. Then, we will add and fuse the attention weights obtained through CA and SA, and integrate the attention weights generated by CA and SA into the original feature map through multiplication operation. This not only achieves optimization of features in both spatial and channel dimensions but also enhances the model’s sensitivity and robustness to crack features. The formula is as follows:

9 $$AttentionModel(x) = x \odot \, SpatialAttention(x \odot \, ChannelAttention(x)) + x$$

where x is the input feature with a shape of $\left( {B,C,H,W} \right)$, $B$ is the batch size, $C$ is the number of channels, and $H$ and $W$ are the height and width of the feature map, respectively. $ChannelAttention\left( \cdot \right)$ and $SpatialAttention\left( \cdot \right)$ represent channel attention and spatial attention.

#### Channel attention block (CAB)

The channel attention block (CAB) dynamically adjusts the importance of different channels by assigning distinct importance weights to each channel. This enhances the model’s sensitivity to different input channels, enabling it to focus on more useful feature information while suppressing less significant features.

As shown in Fig. 2b, in the implementation of the CAB, we initially process the input feature map through both adaptive average pooling and adaptive max pooling layers. Following this, the pooled features are handled by fully connected layers. Lastly, the outcomes of these processing steps are summed and passed through a Sigmoid function to derive the attention weight for each channel. These weights can be utilized to enhance or suppress the information in different channels of the feature map. The formula is represented as follows:

10 $$ChannelAttention(x) = \sigma (FC(AdaptiveAvgPool(x)) + FC(Adaptive\max Pool(x)))$$

where x is the input feature map with a shape of $\left( {C,H,W} \right)$, where $C$ is the number of channels, and $H$ and $W$ represent the height and width of the feature map, respectively. $AdaptiveAvgPool\left( \cdot \right)$ represents the adaptive average pooling operation, which pools the feature map down into dimension of $\left( {C,1,1} \right)$. $AdaptiveAvgPool\left( \cdot \right)$ represents the adaptive maximum pooling operation, which similarly pools the feature map to a dimension of $\left( {C,1,1} \right)$. $FC\left( \cdot \right)$ stands for a sequence of fully connected layers, comprising two convolutional operations and a Sigmoid activation function. $\sigma \left( \cdot \right)$ is the Sigmoid activation function.

#### Spatial attention block (SAB)

The spatial attention mechanism focuses on spatial position information in the images. By identifying and concentrating on key areas where cracks are located, the module can more effectively segment crack features from complex backgrounds.

As shown in Fig. 2c, in the implementation of spatial attention block (SAB), we first aggregate channel information by calculating the mean and maximum values. Subsequently, a spatial attention map of the same size as the input feature map is generated through convolution and the Sigmoid function. This attention map can be used to weigh the spatial locations of input feature map, thereby enhancing or suppressing information from different spatial positions. The formula is represented as follows:

11 $$SpatialAttention(x) = \sigma (Conv([Avg(x),Max(x)]))$$

where x is the input feature with a typical shape of $\left( {C,H,W} \right)$, in which $C$ represents the number of channels, and $H$ and $W$ represent the height and width of the feature map, respectively. $Avg\left( \cdot \right)$ denotes calculating the average along the channel dimension $C$, resulting in a shape of $\left( {1,H,W} \right)$. $Max\left( \cdot \right)$ indicates finding the maximum value along the channel dimension $C$, also yielding a shape of $\left( {1,H,W} \right)$. $\left[ { \cdot , \cdot } \right]$ represents concatenating the two feature maps along the channel dimension, resulting in a shape of $\left( {2,H,W} \right)$. $Conv\left( \cdot \right)$ stands for a 2D convolution operation applied to the concatenated feature map, outputting a feature map with a shape of $\left( {1,H,W} \right)$. $\sigma \left( \cdot \right)$ is the Sigmoid activation function, used to normalize the output feature map of the convolution to the range of $\left[ {0,1} \right]$, generating the final spatial attention map.

### Feature fusion module (FFM)

**Fig. 3:**
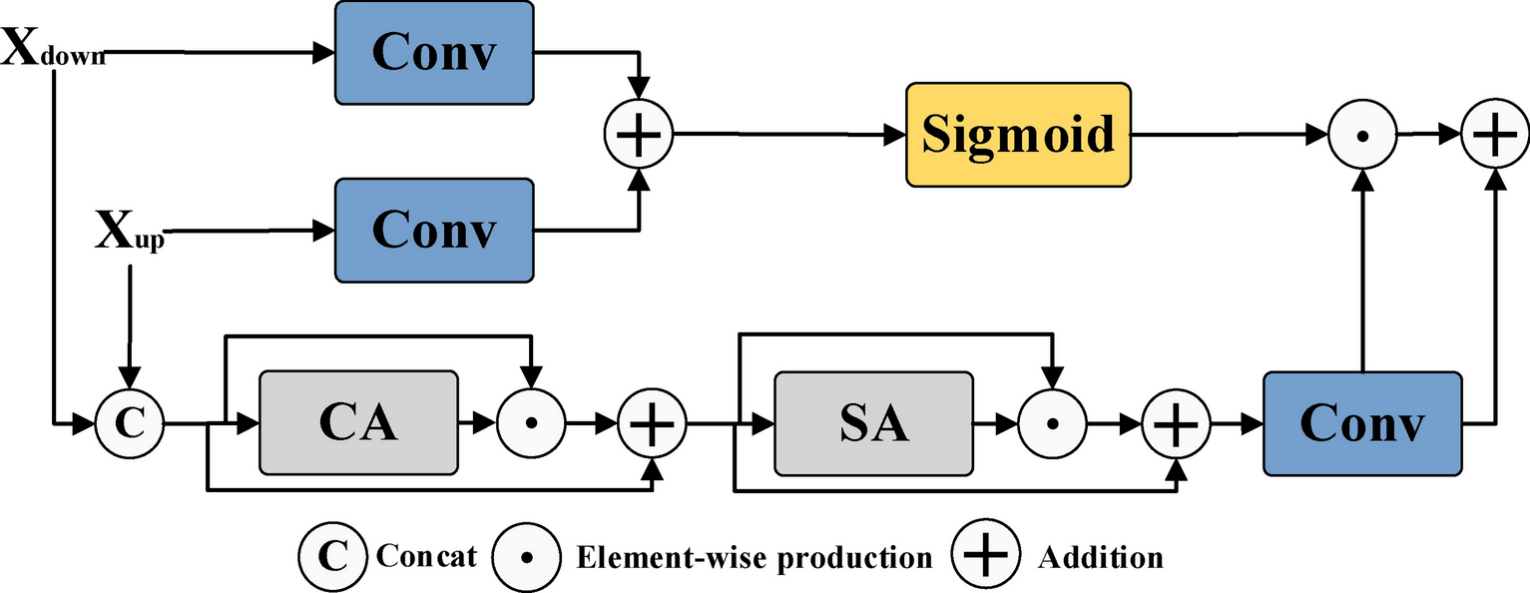
The feature fusion module FFM proposed in this article effectively fuses two input feature maps through convolution and Concat operations to generate more refined feature maps. Meanwhile, with the innovative channel attention (CA) and spatial attention (SA) mechanisms, FFM can accurately focus attention on the regions of interest.

In this study, we designed a feature fusion module to improve the performance of crack image segmentation by fusing feature information from the encoder and decoder. As shown in Fig. 3a, its uniqueness lies in its innovative dual attention mechanism, namely channel attention (CA) and spatial attention (SA), which play a key role in the module.

The fusion module receives two input features: one of which is the feature $X_{down}$ enhanced by the encoder’s attention module, and the other is the feature $X_{up}$ obtained by upsampling through the decoder’s VSS. $X_{down}$ is rich in attention-enhanced feature information, while $X_{up}$ provides further enhanced feature details.

In the fusion process, firstly, $X_{down}$ and $X_{up}$ will extract features through convolutional layers to enhance their feature expression ability. Then, the extracted features are added together and subjected to non-linear processing using the Sigmoid activation function to generate more refined fusion features $X_{S}$.

In order to further enhance the richness and expressive power of the features, we perform channel fusion on $X_{down}$ and $X_{up}$ to generate a new feature $X_{concat}$. Next, we utilize the channel attention mechanism (CA) to process $X_{concat}$, resulting in the feature $X_{CA}$. In this process, the CA mechanism learns and evaluates the importance of each feature channel, adaptively adjusts the weights of each channel to strengthen key features and weaken non-key features. This approach ensures that the model focuses more on the feature channels that are most critical for the crack segmentation task. Subsequently, $X_{CA}$ is processed through the spatial attention mechanism (SA) to capture spatial correlations in the feature map and generate feature $X_{SA}$. The introduction of SA mechanism enables the model to more effectively identify and emphasize key regions, while reducing the influence of backgrounds or irrelevant regions, thereby enhancing the model’s focus on crack regions.

Finally, we pass the SA processed feature $X_{SA}$ through a convolutional layer (Conv) for deep feature extraction, resulting in the feature $X_{o}$. Afterwards, we perform element-wise feature multiplication to fuse $X_{o}$ with the previously generated feature $X_{S}$, and then add the fused feature information to the original $X_{o}$ through an addition operation, ultimately generating the output feature $X_{Fusion}$. The formula is represented as follows:

12 $$x_{S} = \sigma \left( {Conv\left( {x_{down} } \right) + Conv\left( {x_{up} } \right)} \right)$$

13 $$x_{concat} = \left[ {x_{down} ,x_{up} } \right]$$

14 $$x_{CA} = x_{concat} + x_{concat} \odot CA\left( {x_{concat} } \right)$$

15 $$x_{SA} = x_{CA} + x_{CA} \odot SA\left( {x_{CA} } \right)$$

16 $$x_{o} = conv\left( {x_{outputSA} } \right)$$

17 $$x_{Fusion} = x_{S} \odot x_{{\text{o}}} + x_{{\text{o}}}$$

where $\sigma \left( \cdot \right)$ represents the Sigmoid activation function, Conv represents the convolutional operation, $\left[ { \cdot , \cdot } \right]$ indicates concatenation along the channel dimension, $\odot$ signifies element-wise multiplication, $CA\left( \cdot \right)$ and $SA\left( \cdot \right)$ represent channel attention and spatial attention, respectively.

## Experiments

### Datasets

**Fig. 4:**
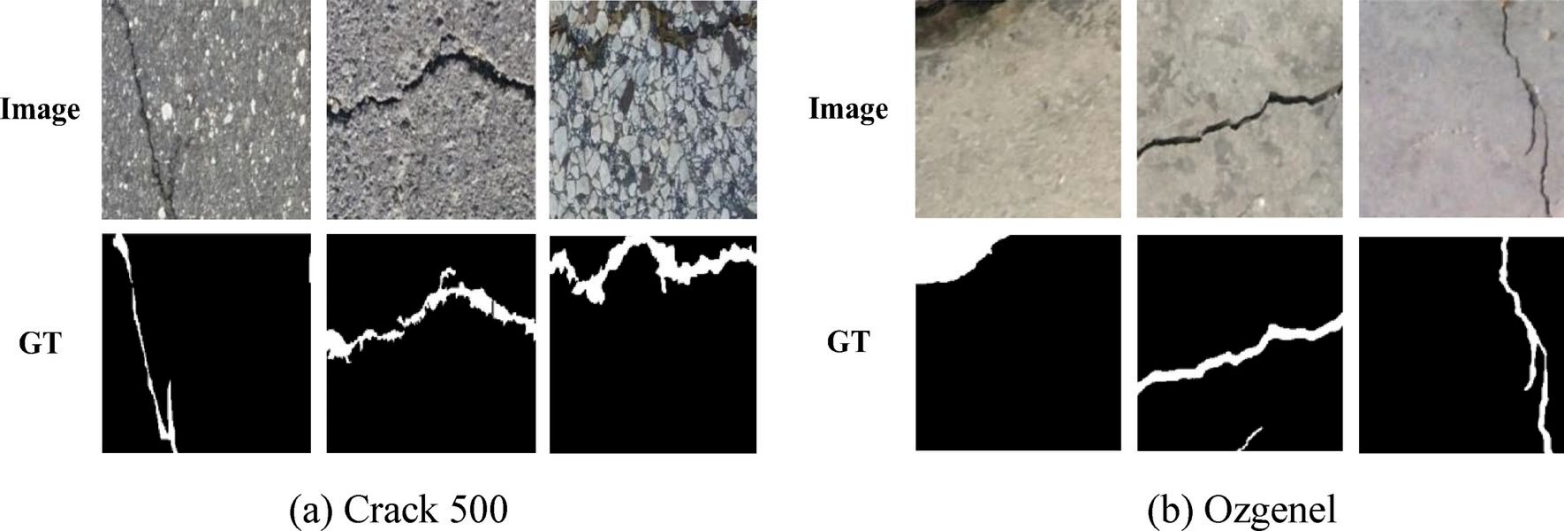
The crack example images of the Crack 500 and Ozgenel datasets in Fig. 5 are divided into two parts: the upper part is the original image, and the lower part is the corresponding crack detection image (GT indicates ground truth).

In this study, to comprehensively and objectively evaluate the performance of our improved VM-UNet model in crack detection tasks, we selected two widely used public crack datasets: Crack500 and Ozgenel. These two datasets cover various complex crack conditions, as shown in Fig. 4. Next, we will introduce these two datasets.

Crack500^35^: This dataset contains 500 images of pavement cracks (with a resolution of 2000 × 1500 pixels), and all these images have been annotated at the pixel level. Due to the limited number of images in this dataset, each image was cropped into 16 non-overlapping regions, retaining areas with more than 1000 pixels. As a result, we obtained 1896 training images and 1124 test images. In this study, to meet the input requirements of the model, we resized all images and their annotations to a uniform size of 448 × 448 pixels.

Ozgenel^36^: This dataset consists of 458 high-resolution images of concrete cracks (with a resolution of 4032 × 3024 pixels) and corresponding annotated masks. The dataset is divided into two categories: images with cracks and images without cracks. In this study, we resized all images into smaller blocks of 448 × 448 pixels to prepare for the evaluation of the dataset. After processing, we obtained a training set containing 1800 images and corresponding masks, as well as a test set containing 454 images and corresponding masks.

As shown in Table 1, the specifications of the two datasets are presented, including the size of input images and the number of images in the training and test sets.

**Table 1:** Details of the two datasets used.

Dataset name	Image size for training	Total number of images	Number of images for train	Number of images for test
Crack500	448 × 448	3020	1896	1124
Ozgenel	448 × 448	2254	1800	454

### Implementation details

This study conducted both the training and testing of the model on Nvidia RTX 3090 GPUs, with the primary software environment consisting of CUDA 11.8 and Python 3.8. The deep learning framework employed was PyTorch version 1.13.0. The Adam optimizer was utilized for training the model, with the training parameters set to a learning rate of 5 × 10^−5^, a weight decay of 0.0001, a batch size of 14, and 100 epochs. To prevent overfitting during the training process, the training dataset was randomly shuffled, and random image augmentation techniques, such as vertical and horizontal flips, were applied.

### Evaluation metrics

In the task of crack segmentation, where the goal is to classify crack and non-crack pixels, the balanced evaluation of segmentation accuracy typically relies on the mean Dice score (mDS) and mean intersection over union (mIoU) as the primary evaluation metrics^37^. Therefore, this study uses mDS and mIoU to evaluate model performance. Their definitions are expressed as follows:

18 $$DS = \frac{{2\left| {P \cap T} \right|}}{\left| P \right| + \left| T \right|}$$

19 $$IoU = \frac{{\left| {P \cap T} \right|}}{{\left| {P \cup T} \right|}}$$

In this context, P represents the pixel map output by the model, and T is the corresponding ground truth pixel map.

Intersection over union (IoU) measures the similarity between the predicted mask and the ground truth segmentation map by comparing their intersection and union areas. The Dice score (DS) evaluates their similarity by calculating the ratio of twice the intersection area to the sum of the areas of both the predicted and ground truth masks. Both evaluation metrics have a scoring range from 0 to 1, where 1 represents a perfect match and 0 indicates no overlap. It is worth noting that when the overlapping region is small, the IoU criterion is relatively stricter, often resulting in lower scores compared to DS. However, as the overlapping area increases, the difference between the two gradually diminishes. Therefore, mIoU is more sensitive to categories with low prediction accuracy, while mDS provides a more comprehensive reflection of the model’s overall segmentation effectiveness across all categories. Importantly, both IoU and DS do not rely on specific pixel classification thresholds during the evaluation process; instead, they consider the entire segmentation region, including overlap at the boundaries. This characteristic makes both metrics fairer and more objective for assessing image segmentation performance, which is why they are widely used.

## Results

### Accuracy comparison

To evaluate and compare the performance of our improved VM-UNet++ model, this study employs established benchmarks represented by various architectures in the field. Specifically, we consider the following representative models: CNN-based UNet^15^ and LinkNet^38^ with EfficientNet^39^ as the backbone (Net-EB7 and LinkNet-EB7); Transformer-based SwinUNet^21^ and SegFormer-B5^11^; CNN-Transformer hybrid designs, namely TransUNet^40^ and DTrC-Net^41^; the efficiently self-attention designed PoolingCrack^42^ and the base model VM-UNet^33^. Through comparative analysis, we aim to validate the performance enhancements achieved by our modified VM-UNet++. Next, we will further highlight the advantages and practical application value of our model in crack detection tasks through model comparison analysis.

We investigated the results of these models (see Table 2) and compared them with our improved VM-UNet, which stands out due to its unique dual attention and feature fusion modules. This design significantly enhances the model’s segmentation performance. As shown in Table 2, VM-UNet++ achieved substantial improvements in both the mDS and mIoU metrics. Specifically, on the Crack500 and Ozgenel datasets, ours model improved mDS by 3.2%, and mIoU showed a significant increase of 4.6–6.2%. Table 2 provides a detailed comparison of the accuracy of different network architectures on the two datasets. It is evident from the table that, compared to other leading models such as UNet-EB7, LinkNet-EB7, and TransUNet, our VM-UNet++ demonstrates superior performance in both mDS and mIoU metrics, while maintaining a relatively low parameter count. Notably, on the Ozgenel dataset, VM-UNet++ achieved an mDS of 90.3% and an outstanding mIoU of 82.3%, which clearly demonstrates the remarkable performance of the improved VM-UNet model in handling complex image segmentation tasks.

**Table 2:** Comparison of accuracy of different network structures on the dataset.

Model	Param (M)	Crack500		Ozgenel	
		mDS (%)	mIoU (%)	mDS (%)	mIoU (%)
UNet-EB7^15^	67	69.9	55.7	84.1	77.3
LinkNet-EB7^38^	64	69.9	55.6	84.6	78.1
TransUNet^40^	106	70.2	56.0	85.3	79.2
SwinUNet^21^	42	68.1	53.3	83.3	76.1
SegFormer-B5^11^	85	70.7	56.5	84.9	78.6
DTrC-Net^41^	42	67.5	53.3	84.7	78.3
PoolingCrack^42^	32	70.6	56.4	85.7	79.7
VM-UNet^33^	**27**	70.3	56.0	85.7	79.4
VM-UNet++ (Ours)	55	**73.4**↑	**57.9**↑	**90.3**↑	**82.3**↑

Significant values are in [bold].

**Fig. 5:**
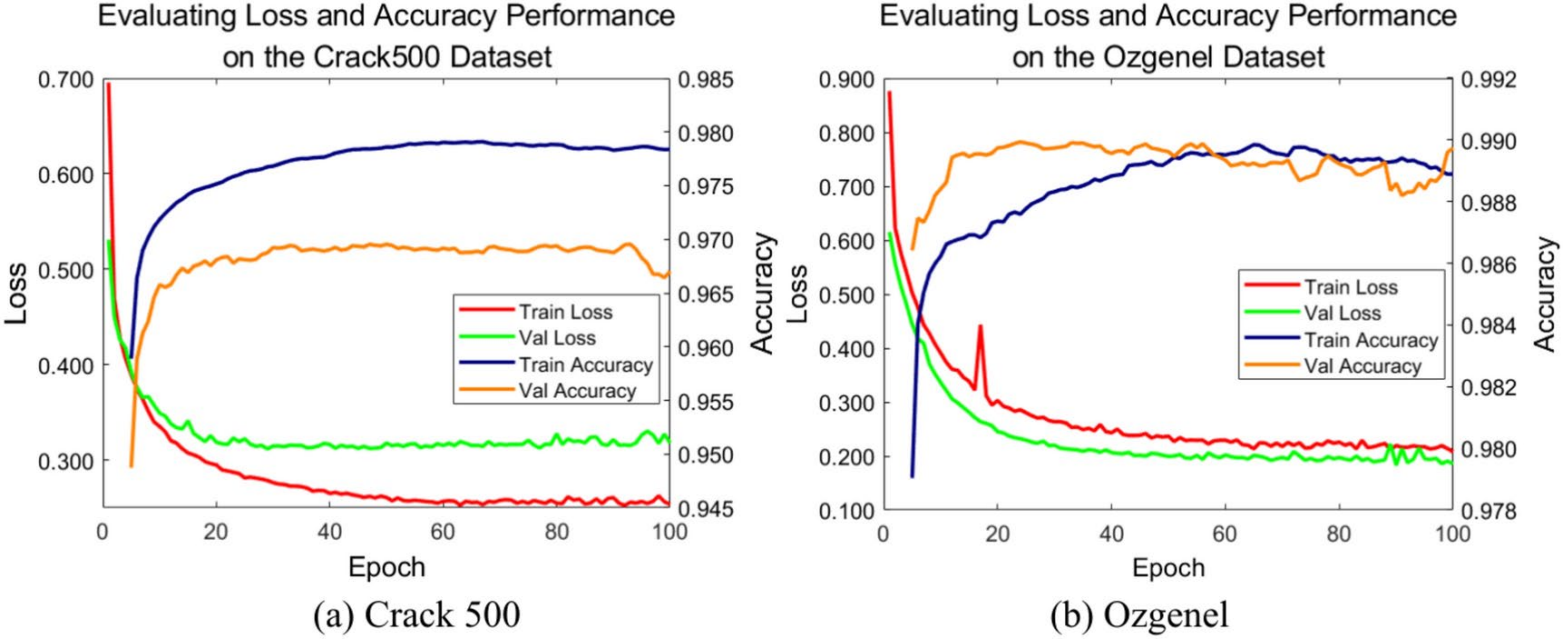
Evaluate the loss and accuracy performance of two datasets: (**a**) Crack 500 and (**b**) Ozgenel.

Figure 5 illustrates the experimental results of VM-UNet++ on the Crack500 and Ozgenel datasets. It can be observed that VM-UNet++ performs remarkably well on both training and validation sets, with the loss rapidly converging and accuracy steadily improving, demonstrating its strong generalization ability. As shown in Fig. 5a, on the Crack500 dataset, the training loss stabilizes after the 20th epoch, and the validation loss follows a similar trend. The training and validation accuracies reach approximately 0.985 and 0.980, respectively, after the 80th epoch, with no significant overfitting observed. Similarly, as shown in Fig. 5b, on the Ozgenel dataset, the training loss stabilizes after the 15th epoch, while the validation loss exhibits some fluctuations initially but gradually decreases. The training and validation accuracies stabilize at approximately 0.990 and 0.988, respectively. These results indicate that the VM-UNet++ effectively learns features from different datasets and achieves consistent and robust performance on the validation sets, aligning closely with the training results.

**Fig. 6:**
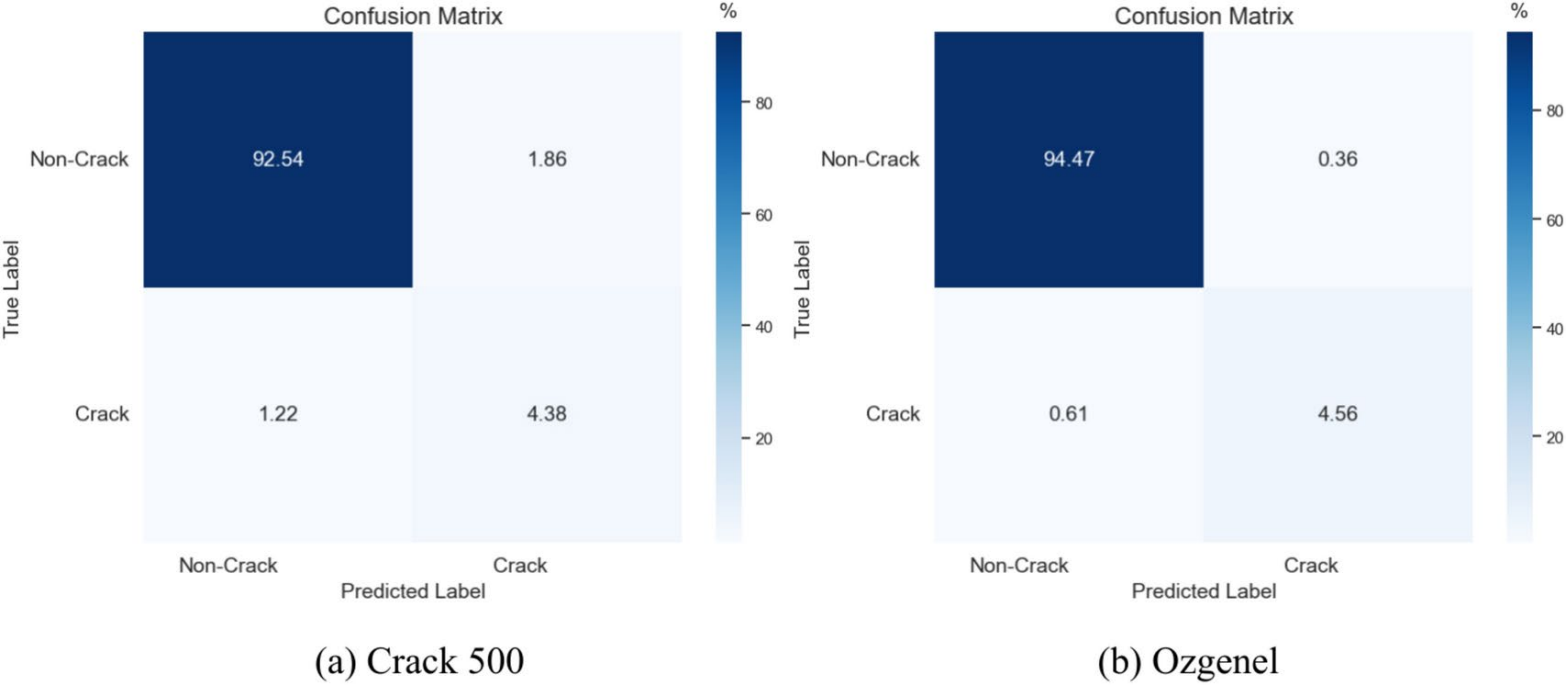
Confusion matrix for Crack500 and Ozgenel datasets.

As shown in Fig. 6, VM-UNet++ performs excellently in the task of crack detection for both the Crack500 and Ozgenel datasets through the confusion matrix analysis. For the Crack500 data set, the true case rate (TN) is as high as 92.54%, the false positive case rate (FP) is as low as 1.86%, and the true case rate (TP) is as high as 4.38% in the recognition of crack images, which strongly proves that VM-UNet++ has a certain accuracy in the recognition of crack images. In the detection of Ozgenel data set, the true case rate (TN) of the model to identify non-crack images is 94.47%, the false positive case rate (FP) is 0.36%, and the true case rate (TP) of the model to identify crack images is 4.56%, which also clearly reflects the reliability of the model in the crack detection scene. The excellent performance of the model on these two data sets fully demonstrates its strong application value and potential in the field of fracture detection.

**Fig. 7:**
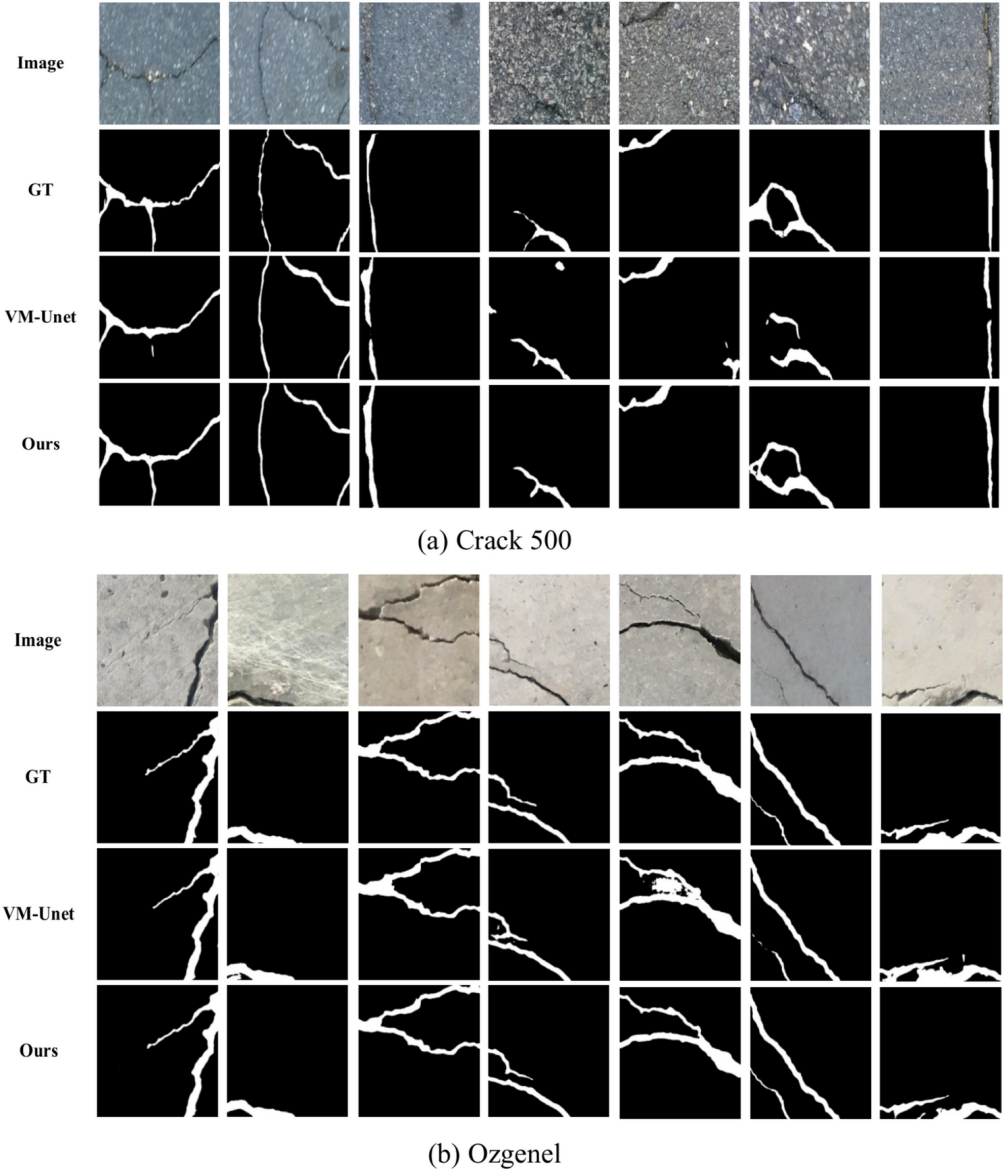
Prediction results on the two datasets: (**a**) Crack 500 and (**b**) Ozgenel.

Figure 7 shows the predictions for both data sets. On the Crack500 dataset, VM-UNet++ demonstrates the ability to generate clear and continuous crack segmentation maps in most cases, significantly better than VM-UNet. As shown in Fig. 7a, VM-UNet exhibits several significant shortcomings, such as the lack of crack pixels in the first, second, sixth, and seventh columns, and the misclassification of some background noise as cracks in the fourth and fifth columns. These problems lead to unstable crack profile delineation. In contrast, the modified VM-UNet++ produces more consistent crack profiles, showing greater robustness and accuracy. It enables a more reliable classification of crack pixels and provides a more accurate representation of the actual crack profile. Similarly, on the Ozgenel dataset, VM-UNet++ also shows superior result. As shown in Fig. 7b, the crack segmentation map generated by VM-UNet++ is more complete and the crack boundary is clearer than that generated by VM-UNet. These results further validate the high accuracy and robustness of VM-UNet++ in the task of crack image segmentation.

### Efficiency comparison

**Fig. 8:**
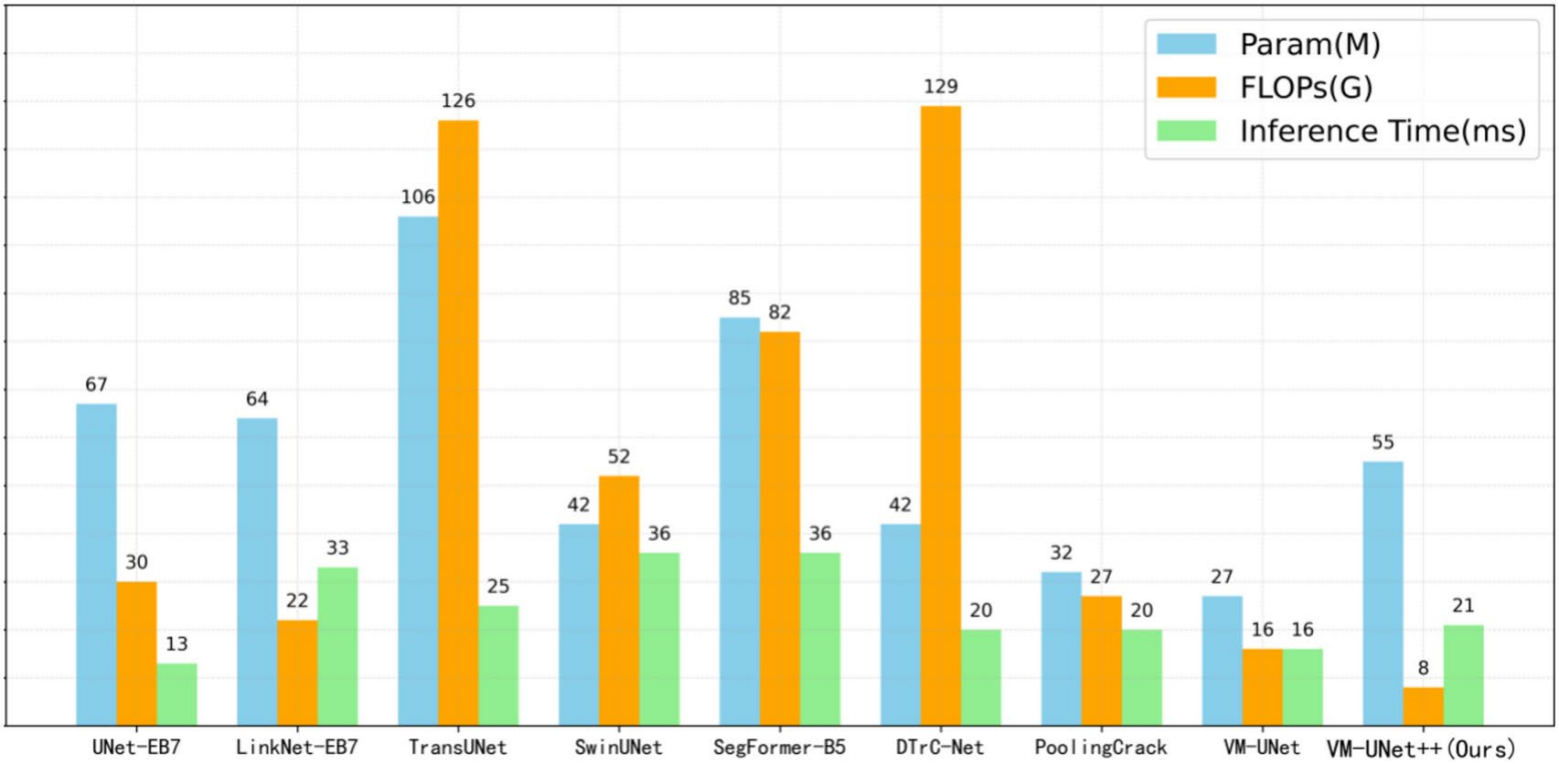
Comparison of model efficiency for processing 448 × 448 resolution images.

To evaluate the efficiency of the model, we selected three key metrics: model parameters, floating-point operations (FLOPs), and inference time. The number of parameters is used to measure the complexity of the model, the number of floating-point operations assesses the computational workload, and the inference time reflects the duration required for the model to make predictions. The relevant evaluation results can be found in Fig. 8.

In this study, we present a comprehensive comparison of VM-UNet++ with other state-of-the-art segmentation models. Compared to other models, VM-UNet++ reduces the number of parameters, such as a decrease of approximately 17.9% compared to UNet-EB7, and about 48.1% compared to TransUNet. However, it increases by approximately 50.1% when compared to VM-UNet. In terms of computational complexity, VM-UNet++ also demonstrates superior performance, with its FLOPs significantly lower than those of other models. Specifically, it reduces FLOPs by about 73.3% compared to UNet-EB7, and by as much as 93.8% compared to DTrC-Net. When compared to VM-UNet, it achieves a 50% reduction. Although the inference time is not the shortest, VM-UNet++ reduces inference time by only about 15 ms compared to other high-performance models such as SwinUNet and SegFormer-B5. While the inference time of VM-UNet++ is approximately 5 ms longer compared to VM-UNet’s 16 ms, this increase is entirely acceptable when considering the significant optimizations in parameters and FLOPs relative to other models.

### Ablation studies

To evaluate the actual effectiveness of our proposed dual attention module and feature fusion module within VM-UNet, we conducted four ablation experiments, as presented in Table 3. Our network architecture primarily comprises three components: the VM-UNet, the dual attention module, and the feature fusion module. The first row of Table 3 illustrates our base model, VM-UNet. The second row demonstrates the impact of incorporating the dual attention module. The third row reflects the results obtained after integrating the feature fusion module. A comparison of the second and third rows in Table 3 clearly indicates that both the dual attention module and the feature fusion module significantly enhance the model’s segmentation performance. Notably, the combined utilization of the dual attention and feature fusion modules in the fourth row yields the best results in terms of mDS and mIoU metrics.

**Table 3:** Research on the ablation of dual attention and feature fusion.

Attention block	Feature fusion module	Crack500		Ozgenel	
		mDS (%)	mIoU (%)	mDS (%)	mIoU (%)
–	–	70.3	56.0	85.7	79.4
√	–	73.0	57.5	89.8	81.5
–	√	73.1	57.6	89.8	81.5
√	√	73.4	57.9	90.3	82.3

## Conclusions

In the research presented in this paper, the dual attention mechanism and feature fusion module have demonstrated exceptional performance in image segmentation tasks. These modules not only improve the accuracy and precision of segmentation but also enhance the robustness and generalization ability of the model. This innovative design brings new breakthroughs to the field of image segmentation and lays a solid foundation and valuable reference for subsequent related research.

## Future work

Regarding our future work plans will focus on several key directions. First, we plan to further explore the performance of the VM-UNet model in handling higher-resolution images. High-resolution image processing has become a critical area of development in the industry, and we anticipate that VM-UNet will demonstrate its unique advantages in this field. Additionally, we aim to investigate the potential application of Mamba technology in other image processing tasks, such as object detection. Crack detection and object detection are closely related in image processing, and we expect that Mamba’s powerful capabilities will lead to innovative advancements in tasks such as crack detection and segmentation. Through continuous research and practical applications, we aim to establish Mamba as a robust tool in the field of image processing, contributing to the growth and development of this domain.

## Data Availability

The datasets generated and/or analyzed during the current study are not publicly available due confidentiality requirements of the school but are available from the corresponding author on reasonable request.
